# Exploring the Epigenetic Landscape of Spermatozoa: Impact of Oxidative Stress and Antioxidant Supplementation on DNA Methylation and Hydroxymethylation

**DOI:** 10.3390/antiox13121520

**Published:** 2024-12-12

**Authors:** Elisa Hug, Yoan Renaud, Rachel Guiton, Mehdi Ben Sassi, Charles Marcaillou, Aron Moazamian, Parviz Gharagozloo, Joël R. Drevet, Fabrice Saez

**Affiliations:** 1 GReD Institute, Université Clermont Auvergne, Faculté de Médecine, CRBC, 28 Place Henri Dunant, 63001 Clermont-Ferrand, Francearon.moazamian@celloxess.com (A.M.); 2 EVALSEM, Clermont Auvergne Innovation, Université Clermont Auvergne, Faculté de Médecine, CRBC, 28 Place Henri Dunant, 63001 Clermont-Ferrand, France; 3 IntegraGen SA, Génopole Campus 1—Bât. 8, 5 Rue de Henri Desbruères, 91000 Evry, France; 4 Centre de Recherche des Cordeliers, INSERM UMRS1138, CNRS SNC 5096, Sorbonne Université, Université de Paris, 75006 Paris, France; 5 CellOxess LLC, Ewing, NJ 08638, USA

**Keywords:** male fertility, epigenetic, antioxidant, DNA methylation, DNA hydroxymethylation

## Abstract

Reproductive success is dependent on gamete integrity, and oxidative stress alters male nuclei, meaning that no DNA repair is possible due to chromatin compaction. The composition of sperm makes it highly sensitive to reactive oxygen species (ROS) but, at the same time, ROS are needed for sperm physiology. Over the past 30 years, much attention has been paid to the consequences of oxidative stress on sperm properties and the protective effects of antioxidant formulations to help fertility. Spermatozoa also carry epigenetic marks, critical for embryo development and the health of offspring. As DNA oxidative damage may disturb the sperm epigenome, we used an established mouse model of post-testicular sperm DNA oxidation to investigate sperm DNA methylation and hydroxymethylation. We also analyzed the potential corrective effect of oral antioxidant supplementation, proven to reduce sperm DNA oxidative damage, on sperm DNA methyl/hydroxymethyl marks. We show that sperm DNA oxidation is associated with a significant increase in overall hydroxymethylation. Oral antioxidant supplementation led to unexpected mild epigenetic changes. Antioxidant supplementation should not be proposed without proper clinical evaluation as it may alter sperm epigenetic marks, leading to a risk of paternally inherited epigenetic alterations.

## 1. Introduction

The mammalian spermatozoon is a unique cell in both its structure and function. The genetic material carried by the male gamete is also unique in its organization via a long and complex transformation process that spans spermatogenesis and post-testicular maturation steps (as reviewed in [1]). During this process, most histones are replaced by protamines, enabling a higher degree of nuclear compaction, around seven times greater than that of somatic cells [2]. The utility of this process is to render sperm nuclei as resistant as possible to mutational attacks, e.g., radiation or oxidative stress, but the consequence is that the genome is inactive, making spermatozoa dependent on their environment for protection and further maturation. The sperm nucleus is specifically packaged; however, it carries the same epigenetic marks found in somatic cells, i.e., histone post-translational modifications (PTMs), DNA methylation, and small non-coding RNAs (sncRNAs; reviewed in [3]).

In the literature, modifications of the three types of sperm epigenetic marks have been related to the intergenerational or transgenerational transmission of metabolic or psychological disorders. For example, metabolic disorders trigger sperm sncRNA pattern modifications [4,5], alteration of histone H3 lysine 4 trimethylation in genes related to metabolic processes [6], or DNA methylation [7]. Consistent with this, the epigenetic marks carried by male gametes are involved in regulating embryo development, as shown for small RNAs in mice ([8]; reviewed in [9]), histone PTMs, and DNA methylation (reviewed in [10]). Studying the sperm epigenome is thus of primary interest to better understand non-genetic inherited disorders caused by paternal exposure, a concept known as “paternal origins of health and disease” (POHaD).

Epigenetic mark acquisition in spermatozoa is a complex process, with many different molecular players depending on the type of mark involved. We will focus on DNA cytosine methylation and hydroxymethylation; readers can find information on histone PTMs and sncRNAs in the literature [10,11]. The sperm DNA methylation profile is progressively acquired during spermatogenesis, reaching around 70% of total DNA in mice and humans [12,13]. The mechanisms of sperm DNA methylation during spermatogenesis, although usually studied alone, involve interaction with histone PTMs (as reviewed in [10]). Classical molecular actors, e.g., DNMT3A, DNMT3c, and DNMT3L, regulate the progression of DNA methylation during spermatogenesis (as reviewed in [14]); however, this will not be described in more detail here, as the current study focused on the effect of post-testicular oxidative stress on sperm DNA methylation. The methylation status of mature spermatozoa is essential for fertility as it can affect embryo development and offspring viability. The underlying mechanisms are not yet understood; the paternal (and maternal) genome must undergo two demethylation steps, the first occurring at the zygote stage and the second in primordial germ cells (as reviewed in [14]). However, certain regions must resist reprogramming to allow proper embryo development, e.g., imprinted genes and transposable elements, suggesting that altering the methylation pattern of sperm DNA could be detrimental (as reviewed in [15]).

During the first demethylation wave in human and mouse zygotes, the paternal genome is actively demethylated by TET3 (ten-eleven translocase 3) hydroxylase, which produces 5-hydroxymethylcytosine (5hmC) from 5-methylcytosine (5mC) [16,17,18]. However, it is not clear whether 5hmC production is related to the demethylation process or if it results from de novo or maintenance methylation [19,20]. It has been questioned whether 5hmC might have functions other than that of a demethylation intermediate, since certain hyper-hydroxymethylated differentially methylated regions (hm-DMRs) are conserved from the male pronucleus to post-implantation stages [20]. Overall, the (hydroxy)-methylation pattern of male gametes appears to be a key element for proper embryo development and offspring health and may be altered under different pathophysiological conditions. A common feature in situations where sperm epigenetic marks can be altered is the creation of oxidative stress in the male genital sphere, as demonstrated in men exposed to bisphenol A [21] during long-term exposure to air pollution [22], or bovine spermatozoa treated in vitro with H_2_O_2_ [23]. Surprisingly, most, if not all, studies have focused on ejaculated or epididymal spermatozoa, yet they have consistently concluded that the altered parameters originated from dysfunctions in the testis. We previously demonstrated that male mice lacking major epididymal luminal antioxidant activity developed sperm DNA hyperoxidation, detected by a dramatic increase in the modified base 8-OHdG in sperm stored in the cauda epididymidis [24,25]. Therefore, post-testicular oxidative stress can oxidize sperm DNA, so we investigated whether it could also alter DNA hydroxymethylation and methylation levels and patterns. We also studied the effect of a specific oral antioxidant supplementation—which has previously been shown in mice to limit the increase in sperm nuclear 8-OHdG [26]—on these epigenetic marks.

## 2. Materials and Methods

### 2.1. Animals and Tissue Sampling

Eight- to ten-month-old male wild-type (WT), *Gpx5^−/−^* (knock-out; KO), and *snGpx4^−/−^; Gpx5^−/−^* (double knock-out; DKO) mice were used. The KO mouse strain is the *Gpx5^−/−^* strain, GPX5 being a glutathione peroxidase specific to the epididymis, and no testis-related disturbance was noticed, the males being fully fertile. The DKO strain is the *snGpx4^−/−^; Gpx5^−/−^* strain, snGPX4 is the sperm nucleus GPX4, an enzyme produced during late spermatogenesis but only active during the epididymal descent to increase chromatin condensation by creating disulfide bridges between protamine thiols. Animals were housed in polypropylene cages (1–6 animals/cage following French regulatory procedures on animal experimentation) under a 12/12-h, light/dark cycle, and acclimated in a controlled environment (room temperature (RT) 24 °C, relative humidity 40–50%, frequent ventilation). Animals were fed a basal diet (Global-diet, 2016S, Harlan, Gannat, France) ad libitum and had free access to water (daily water consumption was visually monitored for classic signs of dehydration to assess treatment intake).

WT, KO, and DKO mice were exposed to oral antioxidant (AOX) supplementation for 14 days (Fertilix^®^, CellOxess, Ewing, NJ, USA, see supplemental Figure S5; this was chosen preferentially as it was shown earlier to efficiently protect KO mouse sperm DNA from oxidative damage [26]) administered via dilution in drinking water. This study was approved by the Comité Régional d’Ethique pour l’Expérimentation Animale (CEMEA-Auvergne) and the French Ministry of Higher Education and Research (APAFIS #33605-2021101917156568v4). Mice were euthanized by cervical dislocation, the epididymides were removed, and the cauda region transferred to a glass dish containing 500 µL of M2 medium (Sigma-Merck) for sperm retrieval. For spermatozoa recovery, cauda epididymides were squeezed with forceps and then punctured several times with a 26G needle. After a 10-min incubation at 37 °C to allow sperm dispersion, these preparations were washed with 500 µL of M2 medium to obtain a final total volume of 1 mL. The sperm count was determined using a Malassez hemocytometer.

### 2.2. DNA Slot Blot Analysis

DNA extraction: sperm genomic DNA was extracted as previously described [19]. Briefly, the collected spermatozoa were centrifuged for 3 min at 1100× *g*, resuspended in 200 μL of solution A (75 mM NaCl and 25 mM EDTA) and 200 μL of solution B (10 mM Tris-HCl pH 8, 10 mM EDTA, 2% SDS, 80 mM DTT) complemented with 1 μg of RNAse A (Qiagen, Courtaboeuf, France), and incubated for 1 h at 37 °C. Next, 100 μg of Proteinase K (Sigma-Aldrich, Saint Quentin Fallavier, France) per 0.5 mL of solution A+B was added and incubated overnight at 55 °C. Sperm genomic DNA was further purified using phenol:chloroform:isoamyl alcohol extraction followed by ethanol precipitation. DNA pellets were resuspended in RNAse/DNAse-free distilled water (Invitrogen, Illkirch, France).

Slot blot protocol: DNA (50 ng) was denatured with 20 μL of NaOH 0.1 M at 95 °C for 10 min. Then, 20 μL of 1M NH_4_OAc solution was added before heat shocking on ice. The solution was diluted in RNAse/DNAse-free distilled water. A nylon membrane was moistened in RNAse/DNAse-free distilled water before DNA samples were deposited. After air-drying, the DNA was cross-linked to the membrane by UV-light exposure. The membrane was rehydrated in TBS-Tween (0.1%) and saturated in TBS-Tween (0.1%)–5% milk for 1 h at RT. For 5mC slot blots, the primary antibody (clone 33D3 at 1/200 in saturating solution, C15200081, Diagenode) was incubated for 2 h at RT. For 5hmC slot blots, the primary antibody (5hmC (pAb) at 1/400 in saturating solution, 39769, Active Motif) was incubated overnight. After washing, an HRP secondary antibody was added for 1 h at RT. Chemiluminescence detection was performed using ECL Western Blotting Reagents (Bio-Rad, Marnes-la-Coquette, France) integrated into the ChemiDoc imaging system (Bio-Rad). ImageJ software (version 1.54) was used for densitometric analysis.

### 2.3. Immunofluorescence for 5mC and 5hmC Nuclear Localization

Sperm suspensions were fixed with 4% paraformaldehyde in PBS for 30 min at 4 °C and smeared on Superfrost glass slides (Fischer Scientific, Marnes-la-Coquette, France). Nuclear decondensation was achieved by 30-min treatment with 4N HCl at 37 °C, then slides were rinsed twice in PBS and sperm cells were permeabilized in PBS-Triton X-100 0.5% for 30 min at 37 °C. Saturation was performed with a solution of Normal Goat Serum 1%–Bovine Serum Albumin 10% in PBS-Tween 0.1% for 1 h at RT. Antibodies against 5mC (1/100, 33D3, Diagenode, Seraing, Belgium) and 5hmC (1/250, 39769, Active Motif, Paris, France) were incubated overnight at 4 °C after dilution in the saturation solution. Slides were incubated with secondary antibodies: goat antimouse-Alexa 488 for 5mC detection and goat antirabbit-Alexa 488 for 5hmC (Invitrogen). DNA was counterstained with Hoechst 33342 (0.001 mg/mL) for 5 min and slides were mounted in Citifluor AF100 anti-fading solution and Citifluor Tris-MWL 4–88 (ratio 1:9; Electron Microscopy Sciences). Observations were made with a Carl Zeiss Axio Scan.Z1. Digital images were processed with Zen software (Carl Zeiss, Rueil-Malmaison, France).

### 2.4. DNA Sequencing: RREM-Seq for 5mC and RREhM-Seq for 5hmC Analysis

Library preparations, FASTQ file generation, alignment, and methylation calling were performed by IntegraGen SA (Evry, France). For reduced representation enzymatic methyl-sequencing (RREM-seq) library preparation, 200 ng of genomic DNA was combined with 1.0 ng of unmethylated Lambda DNA (New England Biolabs) and 0.1 ng of CpG methylated pUC19 (New England Biolabs). This was then digested with 400 units of MspI (New England Biolabs), in NEB Ultra II buffer (New England Biolabs) in a final volume of 57 μL, for 2 h at 37 °C. Following enzymatic digestion, the resulting DNA fragments underwent end-repair and A-tailing using 15 units of Klenow 3′-5′ exo- (New England Biolabs). Once completed, Illumina universal methylated adapters were ligated to the DNA fragments using the NEB Ultra II ligation module, following the manufacturer’s recommendations. After ligation, DNA fragments were purified and size-selected using a two-step SPRI bead purification method to retain 40 to 400 bp fragments. The cleaned, ligated DNA was eluted in 10 mM Tris pH 8 and the purified product was then converted using the Enzymatic methyl-seq module (New England Biolabs), according to the manufacturer’s instructions. In brief, 5mC was converted to 5hmC using TET2, and subsequently, 5hmC was protected from APOBEC deamination through T4-BGT glycosylation. After glycosylation, libraries were denatured at 85 °C for 10 min in 20% formamide. Unmodified cytosines were then subjected to APOBEC deamination. The converted product underwent nine cycles of PCR amplification using indexing primers and KAPA HiFi Hotstart U+ Ready Mix 2X polymerase in a final volume of 50 μL. The resulting PCR product was purified using a 1.2X SPRI ratio and subsequently size-assessed using capillary electrophoresis on a fragment analyzer. Following quantification via qPCR, libraries were sequenced using an Illumina Novaseq 6000 with paired-end 100-bp reads.

For reduced representation enzymatic hydroxymethyl sequencing (RREhM-seq) library preparation, the process closely resembled the RREM-seq protocol, with some modifications inspired by [27]. A total of 200 ng of DNA was mixed with 1.0 ng of unmethylated Lambda DNA and 0.1 ng of CpG-methylated pUC19. After MspI digestion and Klenow 3′-5′ exo-mediated repair, pyrrolo-dC Illumina adapters were utilized in the ligation step instead of 5mC adapters. During the conversion step with TET2, H_2_O replaced TET2 and Fe^2+^ to prevent 5mC protection. The ensuing product was deaminated, amplified, assessed, and sequenced under conditions identical to those of the RREM-seq libraries.

### 2.5. Bioinformatics and Statistics

RREM and RREhM data analysis: FASTQ files were aligned utilizing BSseeker2 (https://github.com/BSSeeker/BSseeker2, accessed on 1 December 2022) against the GRCm38 (mm10) genome using bowtie2. The conversion efficiency was evaluated by analyzing the results of the lambda and pUC genomes added during library preparation. The mmu-10 index was generated with the RRBS option enabled, selecting 40 to 400 bp fragment sizes. Adapter sequences and the CCGG motif were removed during alignment. Post-alignment methylation calling was conducted to determine methylation levels and coverage at each cytosine position within the library. Overlapping and low-quality sequences were removed, as specified by the chosen options. For the RREM-seq libraries, the methylation signal encompassed both 5mC and 5hmC, while in the RREhM-seq libraries, it pertained solely to 5hmC.

Differential methylation and hydroxymethylation studies were conducted using the Bioconductor R package DSS (Park 2016). We performed DML (differentially (hydroxy)-methylated loci) and DMR detection without smoothing, with a delta of 0.1 and an FDR-adjusted *p*-value ≤ 0.05 using callDML() and callDMR() functions, respectively. The genomic distribution of DML for each comparison was determined using the HOMER suite (http://homer.ucsd.edu/homer, accessed on 25 April 2023) with the annotatePeaks.pl program. Gene ontology (GO) studies used the clusterProfiler R package (https://bioconductor.org/packages/release/bioc/html/clusterProfiler.html, accessed on 25 April 2023). All graphics were generated with the ggplot2 R package (https://cran.r-project.org/web/packages/ggplot2/, accessed on 25 April 2023) and statistical tests were conducted using R and GraphPad PRISM (https://www.graphpad.com, accessed on 25 April 2023).

To investigate co-enrichment between 8-OHdG and 5hmC, we aligned DKO and WT conditions for 8-OHdG detection with bowtie2 against the mm10 genome (references ERX1236390 and SRX738874, respectively, available in Sequence Read Archive (SRA) data). Sequencing data (8-OHdG) were processed to remove PCR duplicates using the samtools-rmdup function. To assess DKO peak enrichment vs. WT, we used macs2 with standard parameters, obtaining 477 peaks. We then generated a bigwig file with the Deeptools bamCompare function to obtain the log2 ratio enrichment between DKO and WT conditions over the genome with a bin size of 100 bp. A boxplot was drawn with package R “ggplot2” for an overview of 8-OHdG enrichment between DKO and WT conditions for each chromosome. Only regions different from zero were considered. To visualize the co-enrichment of 8-OHdG DKO vs. WT on enriched 5hmC loci, we focused on DML (±50 bps) found between DKO and WT for the 5hmC signal and generated a violin plot with the ggplot2 function representing log2 signal enrichment for 8-OHdG in these zones. The same comparison was performed for DML between DKO-AOX and WT.

## 3. Results

### 3.1. Observation and Quantification of DNA Methylation and Hydroxymethylation

**Figure 1:**
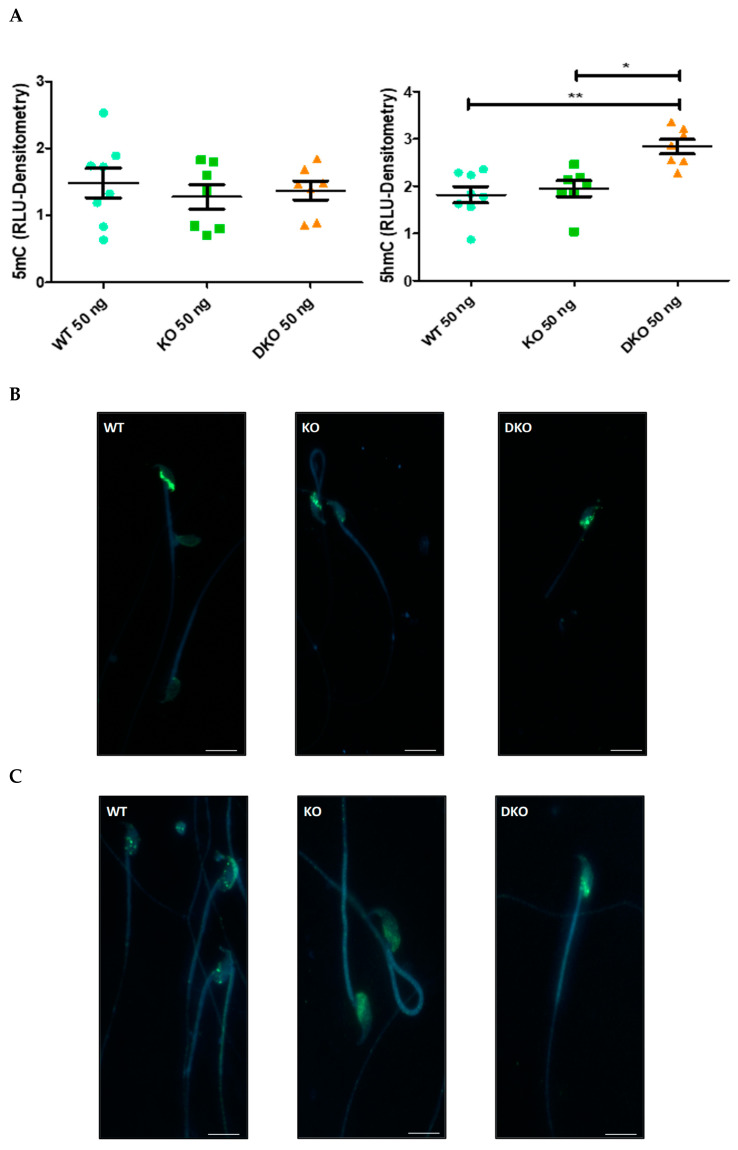
Methylation and hydroxymethylation of the sperm nucleus. (**A**) Global DNA levels of 5mC (**left**) and 5hmC (**right**) determined by slot blot and densitometric analyses. The data represent mean ± S.E.M. of n = 7 individuals per group. * *p* < 0.05, ** *p* < 0.01, using the Mann–Whitney test. 5mC (**B**) and 5hmC (**C**) localization in mouse sperm nuclei determined by immunofluorescence (green). DNA was stained with Hoechst 33342 (blue). The photomicrographs are representative of the staining patterns observed in all individuals tested of the same genotype. Scale bar = 5 µm.

To test whether post-testicular oxidative stress in the two mouse models we have analyzed (KO and DKO) could affect cytosine methylation and hydroxymethylation levels, slot blot experiments were performed on genomic DNA from cauda epididymis spermatozoa (Figure S1 shows representative blots). Densitometric analyses of the slot blots showed no change in the 5mC content detected by this method (Figure 1A, left panel). The 5hmC content of sperm DNA increased significantly in DKO mice, while it remained stable in KO compared with WT mice (*p* < 0.01; Figure 1A, right panel). The 5hmC content was also significantly higher in DKO than in KO mice (*p* < 0.05; Figure 1A). Given that the 8-OHdG content of sperm DNA in DKO mice was also higher than in KO mice due to greater oxidative stress [28], this result suggests a potential link between oxidative stress and DNA hydroxymethylation.

The nuclear localizations of 5mC and 5hmC marks were studied by immunofluorescence (Figure 1B and 1C, respectively). Both marks were present in the basal part of the nucleus, sometimes located in the region covered by the acrosome region for 5hmC in WT mice (Figure 1C, left panel). This distribution correlates with that of 8-OHdG residues, again suggesting a possible link between oxidative stress and DNA hydroxymethylation.

As the increase in 5hmC was specific to DKO mice, these were used in subsequent experiments and compared with the WT strain.

### 3.2. Impact of Oral Antioxidant Supplementation (AOX) on Sperm DNA (Hydroxy)-Methylation

**Figure 2:**
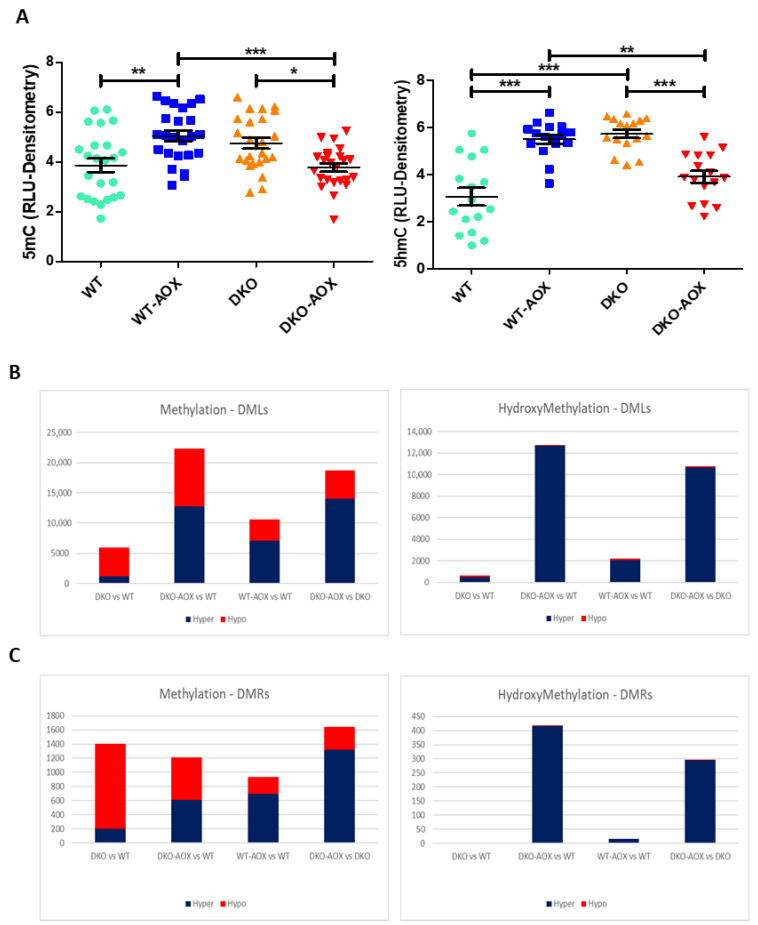
Effect of AOX on the methylation and hydroxymethylation of the sperm nucleus. (**A**) Global DNA levels of 5mC (**left**) and 5hmC (**right**) determined by slot blot and densitometric analyses. The data represent mean ± S.E.M. of 16–24 individuals per group. * *p* < 0.05, ** *p* < 0.01, *** *p* < 0.001 using the Mann–Whitney test. (**B**) Differentially methylated loci (DML) and (**C**) differentially methylated regions (DMR) were obtained after RREM-seq and RREhM-seq data analysis. Data represented were obtained using three biological replicates.

Eight- to ten-month-old male WT and DKO mice were administered AOX in their drinking water at a dose previously shown to reduce the 8-OHdG content of sperm DNA [26]. The treatment lasted for 14 days, corresponding to the epididymal transit period, an organ in which oxidative stress is increased in DKO mice. Levels of 5mC and 5hmC were investigated in genomic DNA extracted from cauda epididymis spermatozoa using the slot blot method (Figure 2A). The 5hmC content of sperm DNA was higher in DKO than in WT mice (*p* < 0.001; Figure 2A, right panel), while the 5mC content was not significantly different, in line with the data presented in Figure 1. Exposure to AOX resulted in a significant reduction in 5hmC levels in DKO sperm DNA (*p* < 0.001; Figure 2A, right panel), suggesting a corrective effect associated with reduced oxidative stress. However, the situation seems to be more complex since there were other effects in both genotypes. AOX caused an increase in 5mC content in WT mice (*p* < 0.01; Figure 2A, left panel) and, conversely, a decrease in DKO mice, also suggesting a potential corrective effect (*p* < 0.05; Figure 2A, right panel). In addition, and rather unexpectedly, WT mice showed an increase in the 5hmC content of their sperm DNA after exposure to AOX (*p* < 0.001; Figure 2A, right panel).

To obtain more precise information on the sequences involved in the methylation and hydroxymethylation changes detected, we performed a modified version of the reduced representation bisulfite sequencing (RRBS) method, RREM-seq. Briefly, in this RRBS variant, DNA is not chemically modified by bisulfite or oxidative bisulfite but by enzymes, enabling methylated and hydroxymethylated cytosine bases to be determined with limited bias. Like RRBS, it is also a reduced representation method and not a whole genome sequencing method since genomic DNA is digested by *MspI* before fragment selection (see the Section 2 for more details).

### 3.3. RREM-Seq for 5mC and 5hmC Characterization

For all samples tested, over 1.5 million CpG sites obtained through RREM-seq or RREhM-seq analysis had a depth between 10× and 100×, allowing for meaningful comparisons (Figure S2A). Most of the cytosines in the CpG context had methylation and hydroxymethylation levels between 0 and 10% (Figure S3). Within this range, there were no obvious differences between groups or individuals. Considering all other methylation and hydroxymethylation levels, represented by 10% intervals, some differences were visible, particularly for 5hmC in AOX-treated mice (Figure S3B). When overall methylation and hydroxymethylation levels were considered (Figure S2B), hyper-hydroxymethylation presented in AOX-treated samples, with a greater increase in DKO individuals. This result correlates with slot blot detection in WT mice but exhibits the opposite trend in DKO mice. Regarding the 5mC content, slight hypermethylation was observed in AOX-treated samples, which correlated with slot blot results for WT; again, this was the opposite in DKO mice.

Levels of 5mC and 5hmC were further analyzed by characterizing the DML seen in Figure 2B, finding hypomethylation and hyper-hydroxymethylation of sperm DNA in DKO compared with WT mice. This agrees with the 5hmC slot blots, except that hypomethylation was not seen with the slot blot approach. AOX increased the number of hypermethylated and hyper-hydroxymethylated sperm DML in WT compared to DKO mice.

**Figure 3:**
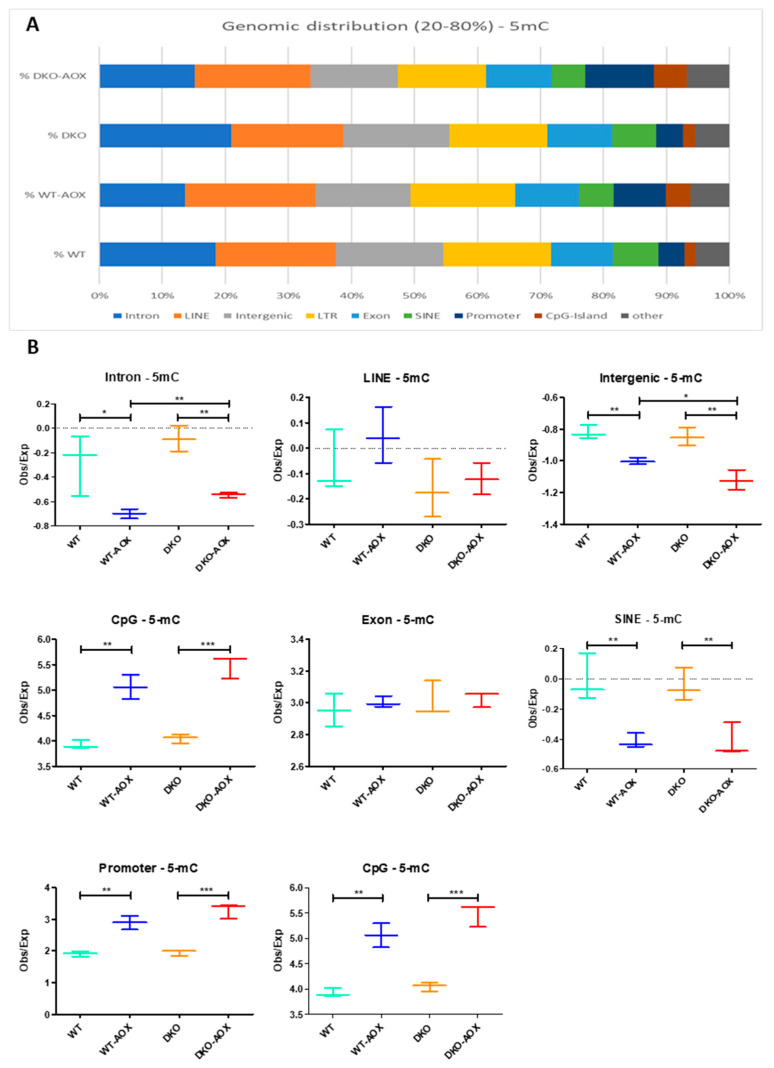
Genomic distribution of 5mC detected by RREM-seq. (**A**) The genomic distribution of 5mC loci is represented on merge replicates for each condition (as % of total 5mC detected) and computed with the Homer suite. (**B**) Statistical analysis of log2 enrichment (observed vs. expected) for each genomic region category is represented in (**A**). Data represent the mean and 5–95th percentile of three biological replicates. * *p* < 0.05, ** *p* < 0.01, *** *p* < 0.001 using the Mann–Whitney test.

**Figure 4:**
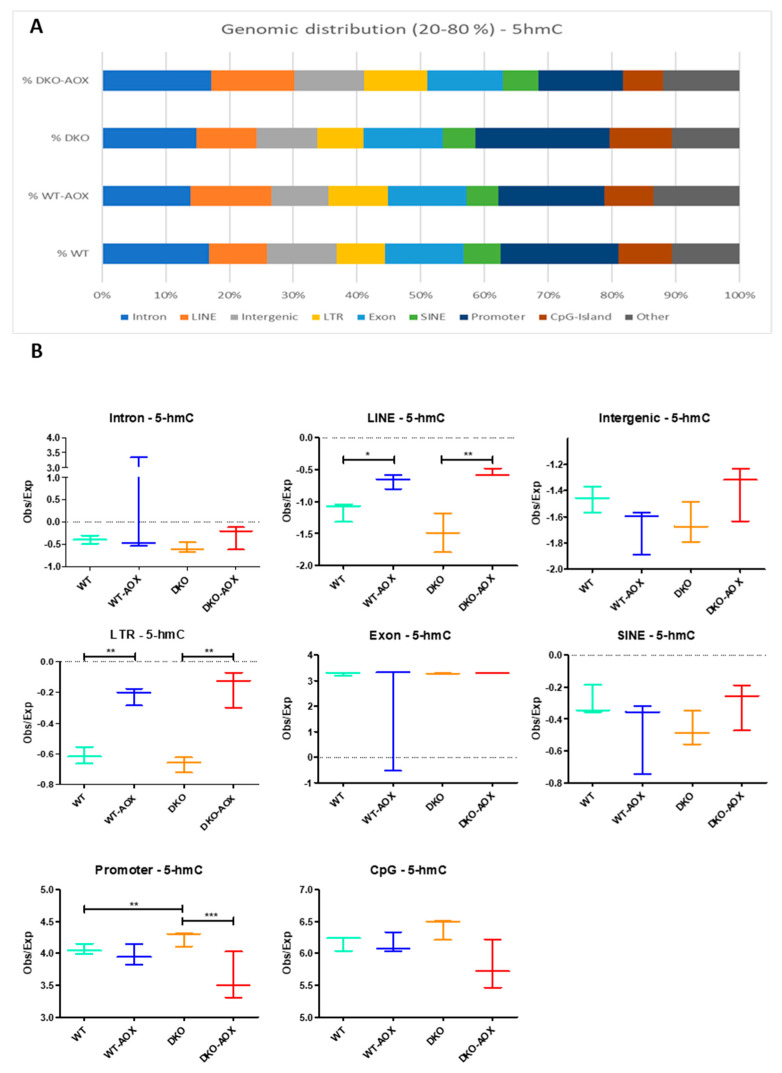
Genomic distribution of 5hmC detected by RRhEM-seq. (**A**) The genomic distribution of 5hmC loci is represented on merge replicates for each condition (as % of total 5hmC detected) and computed with the Homer suite. (**B**) Statistical analysis of log2 enrichment (observed vs. expected) for each genomic region category is represented in (**A**). Data represent the mean and 5–95th percentile of three biological replicates. * *p* < 0.05, ** *p* < 0.01, *** *p* < 0.001 using the Mann–Whitney test.

To advance further, the genomic locations of 5mC and 5hmC were characterized and are represented in Figure 3 and Figure 4, respectively. Here, only DNA bases with methylation or hydroxymethylation levels between 20 and 80% were considered based on prior analyses showing that these bases in spermatozoa are “dynamic” in response to different exposures [29]. Marks of 5mC were more represented in introns, LINEs, intergenic sequences, and LTR regions, and less in exons, SINEs, promoters, and CpG islands (Figure 3A). DML analysis showed overall hypermethylation; however, this was not a general feature as methylation levels decreased significantly after AOX treatment in introns, intergenic regions, and SINEs in both genotypes (Figure 3B). Conversely, hypermethylation was visible in promoters and CpG islands. The 5hmC mark had a different profile to that of 5mC, being mainly in promoters, introns, and exons, with lower percentages in intergenic regions, LINEs, CpG islands, LTRs, and SINEs (Figure 4A). This suggests that cytosine hydroxymethylation does not occur on 5mC bases, which was confirmed by the small overlap between 5mC and 5hmC sites. The hyper-hydroxymethylation revealed by DML analysis occurred mainly in LINEs and LTRs. Promoters were specifically hypo-hydroxymethylated in DKO after AOX treatment.

These changes in 5mC and 5hmC locations in sperm DNA after AOX exposure are not observed if 100% of the sequenced bases are considered and not just dynamic CpGs, as shown in Figure S4. This result confirms that epigenetic modifications triggered by AOX exposure specifically target these dynamic CpGs.

**Figure 5:**
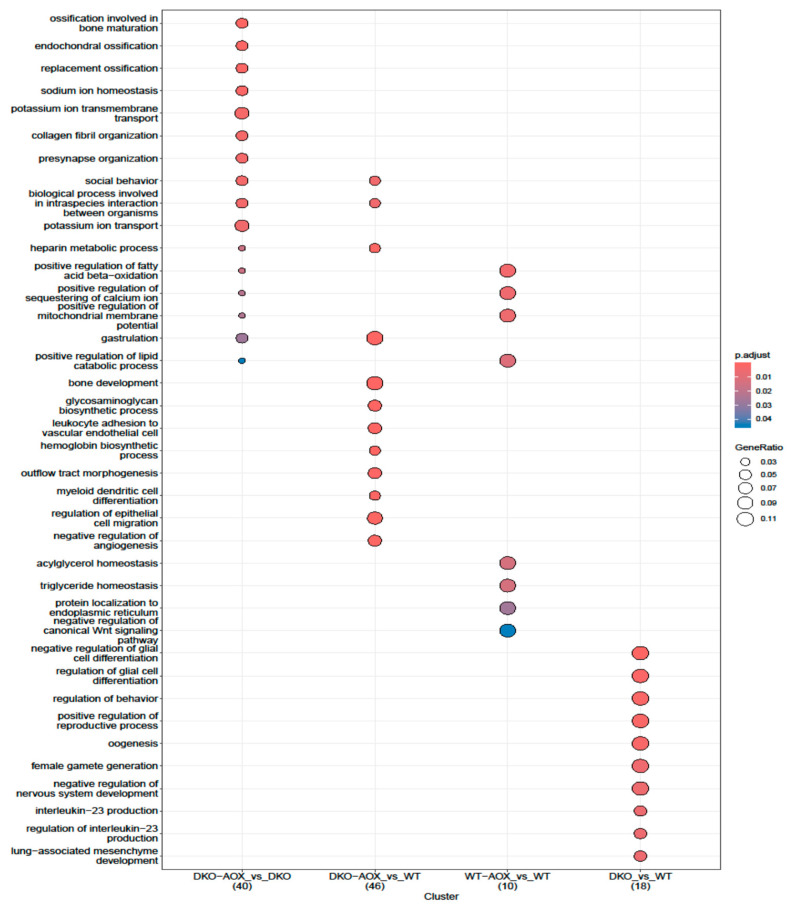
Gene ontology analysis for 5mC DMRs. Biological process GO term enrichment using the enrichGO function from the clusterProfiler R package (organism = “Mm”, *p* ≤ 0.05) for 5mC DMR-associated genes in DKO-AOX vs. DKO, DKO-AOX vs. WT, WT-AOX vs. WT, and DKO vs. WT. The number of genes for each comparison is indicated between brackets.

The potential consequences of these changes were studied by analyzing DMRs, followed by GO, in these regions. As shown in Figure 2C, DMRs could be isolated for 5mC in all comparisons performed, whereas the opposite held for 5hmC; no DMRs were detected when comparing sperm DNA of DKO and WT mice. Only a few DMRs were detected for 5hmC after AOX treatment in WT mice (n = 15); all were hyper-hydroxymethylated. All DMRs, except one, (n = 296) were also hyper-hydroxymethylated when comparing AOX-treated/untreated DKO mice. GO analysis of 5mC DMRs located in promoters in DKO and WT revealed only a few GO categories related to gliogenesis, behavior, reproductive outcomes, and some cell biology processes (Figure 5). When AOX was administered to DKO mice and compared with WT, the genes with DMRs formed part of various processes, including developmental (gastrulation and bone development), metabolic (glycosaminoglycan biosynthesis process, hemoglobin metabolism) and immunity (leukocyte adhesion to vascular endothelial cells, myeloid dendritic cell differentiation). The key finding was that few categories were shared between samples (Figure 5), suggesting that antioxidant supplementation affected the genome randomly, as confirmed by the limited overlap between genotypes. In both comparisons (DKO-AOX vs. DKO and WT-AOX vs. WT), developmental processes were involved in DKO (gastrulation, ossification), while other, mainly metabolic, processes were involved in WT.

**Figure 6:**
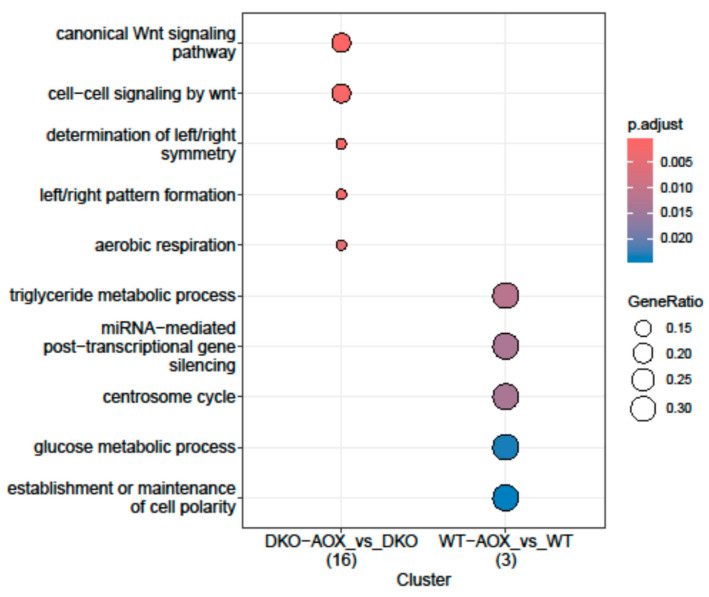
Gene ontology analysis for 5hmC DMR. Biological process GO term enrichment using the enrichGO function from the clusterProfiler R package (organism = “Mm”, *p* ≤ 0.05) for 5hmC DMR-associated genes in DKO-AOX vs. DKO, and WT-AOX vs. WT. The number of genes for each comparison is indicated between brackets.

GO analysis of 5hmC DMRs was only possible in two conditions, comparing each genotype after AOX treatment (Figure 6). The treatment effect was specific to each genotype as no categories were shared. Some categories in DKO were related to embryo development, e.g., the Wnt pathway or symmetry determination.

Unexpectedly, RREM-seq showed that oral antioxidant supplementation—previously proven to protect sperm against DNA oxidation—may alter sperm 5mC and 5hmC marks, albeit very moderately.

### 3.4. Evaluation of a Potential Co-Localization Between 8-OHdG and 5hmC

**Figure 7:**
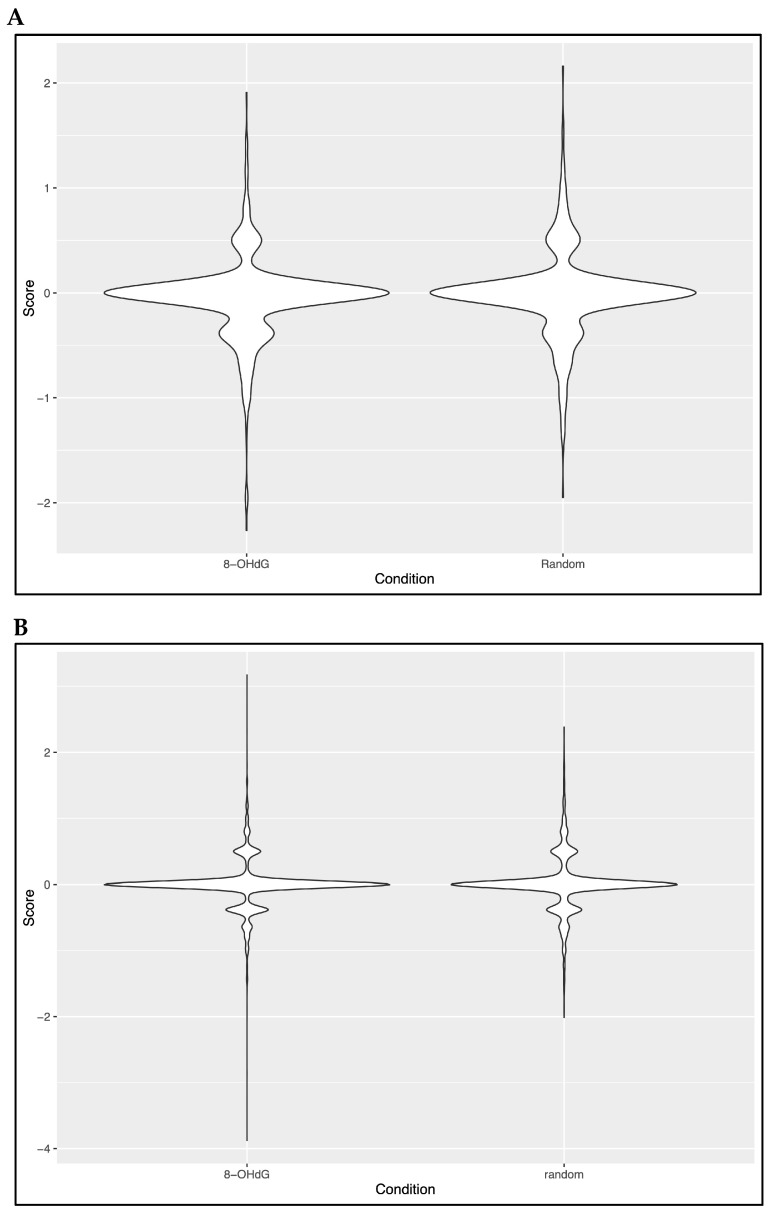
Correlation between 8-OHdG and 5hmC DML. For each 5hmC detected as a DML between DKO and WT (**A**) or DKO-AOX and WT (**B**), we analyzed 8-OHdG enrichment within a ±50-bp interval using previously published data [28]. The log2 8-OHdG enrichment (Score) was plotted using the violin representation, with a score close to zero meaning that no enrichment was detected. The same enrichment analyses were made with 5hmC loci randomly selected throughout the genome (random, right panels in (**A**,**B**)).

The genomic locations of 8-OHdG bases in DKO vs. WT mice were previously published by our group [28]. These data were used to assess the potential co-enrichment of 5hmC and 8-OHdG under our conditions, aligning with our main hypothesis. To this end, 8-OHdG enrichment was considered in a 100-bp zone around each 5hmC DML detected between DKO and WT (Figure 7A) or DKO-AOX and WT (Figure 7B). This enrichment was compared to that of 5hmC loci selected randomly from the genome, excluding DML. In both conditions (5hmC DML or random 5hmC loci), the violin diagrams show that most loci have a score close to zero, meaning that there is no significant 8-OHdG enrichment around 5hmC loci under our experimental conditions. This result seems to confirm that AOX affected the genome randomly, since there is a 5hmC enrichment after AOX treatment.

## 4. Discussion

We addressed two main questions: (1) does epididymal oxidative stress modify the epigenetic marks of cytosine methylation and hydroxymethylation in sperm DNA? and (2) can oral antioxidant supplementation, known to reduce epididymal oxidative stress and correct oxidative damage in sperm DNA [28], also protect these marks? To answer these questions, we used slot blots to quantify 5mC and 5hmC on total genomic DNA, immunocytochemistry to localize 5mC and 5hmC in sperm nuclei, and innovative RREM-seq combined with RRhEM-seq—which can differentiate between 5mC and 5hmC residues—for the detailed characterization of the nucleotides involved. To our knowledge, this study is the first to use the RREM-seq method, in its hydroxylation version, on sperm DNA.

The first important result is that we did not obtain comparable quantitative variations between groups, depending on the method used. These discrepancies may result from the fact that slot blot detections involve the entire genomic DNA, whereas RREM-seq targets only 2–3% of the mouse genome in CpG-rich regions. Furthermore, slot blots are indirect methods involving antibodies, whereas sequencing methods are more direct. Ultimately, the ideal method would be whole genome sequencing, but RREM-seq data are more accessible. As the modifications at base level are the final objective of this work, sequencing remains the reference methodology.

*Effect of genotype on 5mC and 5hmC marks*: Our initial observation with slot blots was that DKO sperm DNA had a higher 5hmC content than WT and KO, with no change in 5mC content (Figure 1A). We attempted to quantitatively measure sperm nuclear 5mc and 5hmC content via mass spectrometry but there were sensitivity issues.

For a more detailed and quantitative approach, we opted for RREM-seq. DML analysis of RREM-seq data revealed an overall hypomethylation of DKO spermatozoa DNA compared with WT (Figure 2B). Also regarding DML, DKO sperm DNA was hyper-hydroxymethylated compared with WT, aligning with the slot blot results, although only 650 DML were characterized (Table S1). Whole genome sequencing could help to determine the extent of DKO sperm DNA hyper-hydroxymethylation.

Detailed analysis of these marks showed a high number of hypomethylated 5mC DMRs in DKO sperm DNA compared with WT (1204 hypomethylated vs. 202 hypermethylated; Table S1); no DMRs were detected for 5hmC marks. The latter observation is unsurprising, as only 650 DML were detected in this comparison, suggesting that 5hmC was not directed to specific regions of sperm DNA but dispersed throughout the genome covered by RREM-seq analysis. GO analysis was performed on the 5mC DMRs characterized between DKO and WT sperm DNA, revealing that developmental pathways were involved, which could impact future embryo development. Nevertheless, the overall GO analysis must be interpreted with caution as, for all the categories shown in Figure 5 and Figure 6, only a few genes were involved. Given that, in DKO mice, 65–70% of sperm exhibit guanosine oxidation (as evidenced by 8-OHdG [25]), a higher level of cytosine hydroxymethylation could be expected. However, this assumes that the rate of cytosine hydroxymethylation is similar to that of guanosine residue oxidation, which is unknown.

*Effect of AOX on each genotype*: Oral supplementation with AOX has previously been shown to reduce DNA 8-OHdG content in transgenic mice, confirming this formulation’s effectiveness in alleviating oxidative stress [26]. Slot blot analysis indicated that, while AOX appeared to normalize 5mC and 5hmC levels in DKO mice, they also induced changes in WT mice, potentially supporting the dual nature of antioxidant micronutrients from a clinical health perspective when taken in excessive doses. While RREM-seq data corroborated these changes, albeit with some variation compared to slot blot results, it additionally revealed persistent modifications in AOX treated DKO mice as well. These modifications, regardless of genetic background, reflect complex mechanisms likely involving both direct and indirect pathways influenced by antioxidant micronutrient formulations. Indirect effects may include the alleviation of oxidative stress through the non-specific prevention of 8-OHdG formation, which affects DNA secondary structures, such as G-quadruplexes [30], and also impacts 8-OHdG’s role as an independent epigenetic regulator [31]. As previously proposed by Moazamian et al., the truncated base excision repair (BER) pathway in sperm allows oxidative stress to induce unique alterations in DNA structure, facilitating both mutagenesis and epigenetic modifications that are related but not necessarily concordant [32]. Direct contributions may involve specific components of the antioxidant formulation. For example, L-5-methyl-tetrahydrofolate (5-MTHF) donates a methyl group to homocysteine, which is converted into methionine and further to S-adenosylmethionine (SAM), the primary methyl donor in the cell [33]. This process has been extensively shown to modify both paternal and maternal methylation patterns [34]. Although the literature largely supports the significant role of folate to influence methylation [34,35,36], recent clinical studies have shown mixed results—likely due to variations in the ingredient form, dose, duration of treatment, or co-administration with other high-dose minerals [34,35,36,37].

To date, the sperm methylome was thought to be mainly acquired during spermatogenesis; epididymal transit was not considered an important contributor [38]. The changes reported here in a pro-oxidant epididymal context (i.e., the transgenic background) and following antioxidant treatment suggest that changes in the sperm nucleus methylome (meC and hmeC) may occur. DNA hypermethylation, when localized to certain genes in somatic cells, has been associated with pathological conditions, e.g., ovarian cancer [39] or renal carcinoma [40]. In male gametes, data are scarce; however, DNA fragmentation and asthenospermia are associated with hypermethylation of the paternally imprinted gene, *IGF-2* [41]. However, it remains to be determined whether DNA hypermethylation is a cause or consequence of these conditions.

In our study, we verified that no DMRs were detected in paternal or maternal imprinted genes. The effect of AOX was more pronounced in DKO spermatozoa nuclei than in WT, which might be related to the less condensed chromatin of DKO spermatozoa, in addition to its greater oxidation, as we previously demonstrated [25]. In mammals, optimal sperm nuclear condensation is achieved in the proximal epididymis via water resorption and oxidative processes [42,43]. Any perturbation of the luminal proximal epididymis environment may impact optimal sperm nuclear compaction. Recently, we showed that the murine proximal caput epididymis is densely irrigated by fenestrated blood vessels, making it highly susceptible to any systemic influences [44]. This may explain why environmental exposures (including oral supplementation) can alter sperm epigenetic marks; an observation already reported for sperm ncRNAs [5,8,45].

The effect of AOX supplementation on each genotype was also examined from a GO perspective. Developmental processes were involved in DKO for 5mC and 5hmC DMRs, while metabolic or cellular processes were revealed in WT. In DKO 5mC DMRs, the gastrulation pathway was highlighted, while the *Wnt* signaling pathway was involved in DKO 5hmC DMRs. This could suggest that these genes are in genomic regions susceptible to DNA oxidation and nuclear compaction. Interestingly, methylation changes in *Wnt* were seen in sperm from obese rats subjected to high fat diets [46] and in mice with vitamin D deficiency [47]. However, in these two situations, hypomethylation was recorded, whereas we studied hypermethylation and hyper-hydroxymethylation. Pyrosequencing could confirm our observations of altered sperm DNA methylation.

The genomic distributions of the 5mC and 5hmC marks described in Figure 3 and Figure 4 revealed different major localizations for each type. Following AOX administration, hypermethylation was mainly located in promoters and CpG islands, irrespective of genotype, while hyper-hydroxymethylation tended to be in LINEs and LTRs, and also for both genotypes. GO analysis of DKO-AOX vs. DKO promoters revealed that 14 of the 25 main pathways impacted for 5mC were linked to developmental or metabolic processes. GO could not be performed on WT promoters but the analysis of coding gene sequences found that 8 of the 25 most involved processes were developmental. This is hardly surprising, considering that sperm nuclear domains sensitive to oxidation are less compacted and rich in persistent nucleosomes, which contain developmental genes (as reviewed in [10]).

Having previously identified the nuclear regions of spermatozoa affected by oxidation in the DKO transgenic context [28], we analyzed the potential for co-enrichment of 5hmC and 8-OHdG. No co-enrichment was detected, suggesting that, under our experimental conditions (DKO and DKO-AOX), 5hmC DML were not located near (within ± 50 bp) 8-OHdG bases, defined as “oxidation hotspots”, in DKO compared to WT mice [28]. In DKO mice, 5hmC DML were few (650; Table S1), and likely too few to reveal any potential co-enrichment. Even when considering the 5hmC DML between DKO-AOX and WT (12749), we found no co-enrichment. This suggests that different regions of the sperm nucleus are targeted in these different situations (sperm nuclei oxidation/decondensation in the DKO background and antioxidant supplementation). It would be interesting to know whether identical results would be obtained using exposures known to affect sperm 5hmC levels and generate oxidative stress. We recently reported that short exposure to low doses of dibutyl phthalate (DBP) and bisphenol AF (BPAF) in WT mice was associated with sperm DNA oxidative damage [48]. Other investigators have shown that mice exposed to similar pollutants presented alterations in the sperm methylome [49,50]. DKO mice could be an interesting model to further study the cumulative effects of endogenous post-testicular oxidative stress and exogenous exposure (pollutants) on sperm DNA oxidation and epigenetic methylation/hydroxymethylation marks, which is a mixed situation typical of multi-exposed humans.

## 5. Conclusions

In conclusion, moderate post-testicular oxidative stress—such as in the model tested here, where sperm nucleus oxidation is not associated with fragmentation—is also associated with a moderate increase in DNA hydroxymethylation, although not at the same CpG dinucleotides. Oral antioxidant supplementation induced some epigenetic modifications in the sperm DNA methylome/hydroxymethylome, although these were minimal and appeared to be logically related to the degree of sperm nucleus compaction. Whether these changes are physiologically relevant requires further study. These results reinforce the notion that, while various exogenous exposures, including antioxidant micronutrients, can influence epigenetic methylation profiles, our understanding of what constitutes “normal” epigenetic health remains incomplete. Even less is known about what might be considered “optimal” epigenetic health, highlighting the need for further research in this area. Additionally, AOX supplementation should not be administered haphazardly without proper clinical evaluation. Both the choice of ingredients and their doses must be carefully controlled to avoid the transgenerational transmission of paternally inherited genetic and epigenetic alterations.

## Data Availability

The datasets used are available at GSE277529.

## Supplementary Materials

The following supporting information can be downloaded at: https://www.mdpi.com/article/10.3390/antiox13121520/s1, Figure S1: Detection of 5mC and 5hmC in sperm DNA extracts; Figure S2: Analysis of individual sequencing data; Figure S3: Cytosine methylation and hydroxymethylation status; Figure S4: Genomic distribution of 5mC and 5hmC detected by RREM-seq; Figure S5: Composition of AOX. Table S1: Comparisons of DML and DMRs for 5mC and 5hmC between different groups.
