# Supplementary material for: Exploring the Epigenetic Landscape of Spermatozoa: Impact of Oxidative Stress and Antioxidant Supplementation on DNA Methylation and Hydroxymethylation
Source: Antioxidants (Basel). 2024 Dec 12;13(12):1520. doi: 10.3390/antiox13121520 (PMC11726892; doi:10.3390/antiox13121520)
Supplement: Supplementary file 1 [file antioxidants-13-01520-s001.zip › antioxidants-3348026-supplementary.pdf]

Supplementary Figures

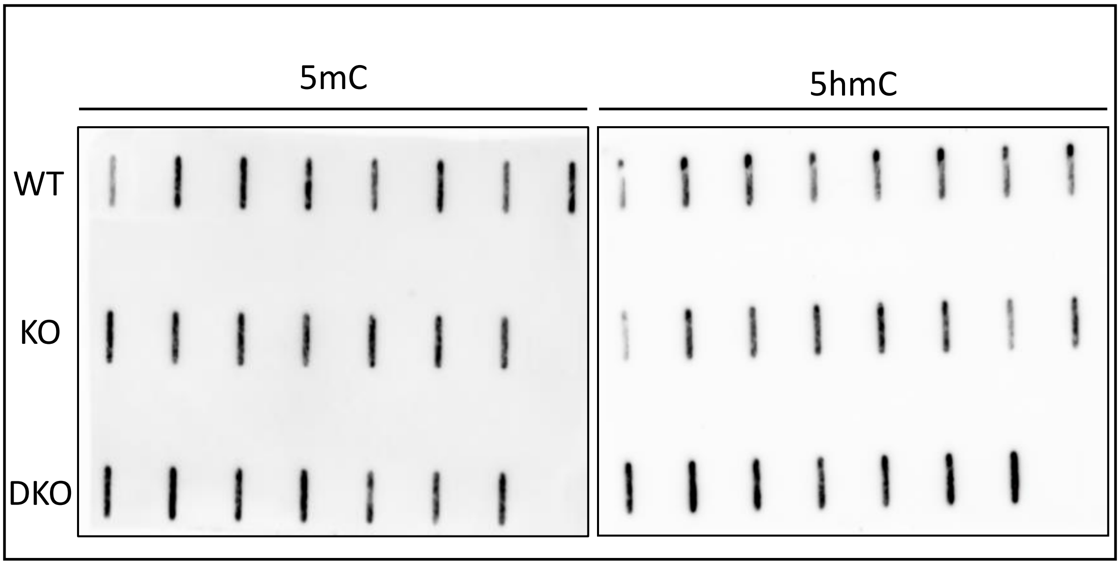

Figure S1. Detection of 5mC and 5hmC on sperm DNA extracts.
After sperm genomic DNA extractions, individual levels of 5mC and 5hmC were detected using the slot-blot method. Each slot represents a single individual. These blots were then analyzed by densitometry to generate the related figures in the paper.

A

	5mC
Sample	Sum **(CpG 10<X<100)**
WT1	2 019 015
WT2	1 903 033
WT3	1 970 895
WT-AOX 1	2 364 198
WT-AOX 2	2 334 782
WT-AOX 3	2 245 083
DKO1	1 599 652
DKO2	1 829 423
DKO3	2 064 465
DKO-AOX 1	2 313 588
DKO-AOX 2	2 313 695
DKO-AOX 3	2 363 527

	5hmC
Sample	Sum **(CpG 10<X<100)**
WT1	2 311 783
WT2	2 214 386
WT3	2 258 522
WT-AOX1	2 157 444
WT-AOX 2	2 226 542
WT-AOX 3	2 166 152
DKO1	2 163 144
DKO2	2 200 647
DKO3	2 230 489
DKO-AOX 1	2 150 322
DKO-AOX 2	2 270 851
DKO-AOX 3	2 301 461

B

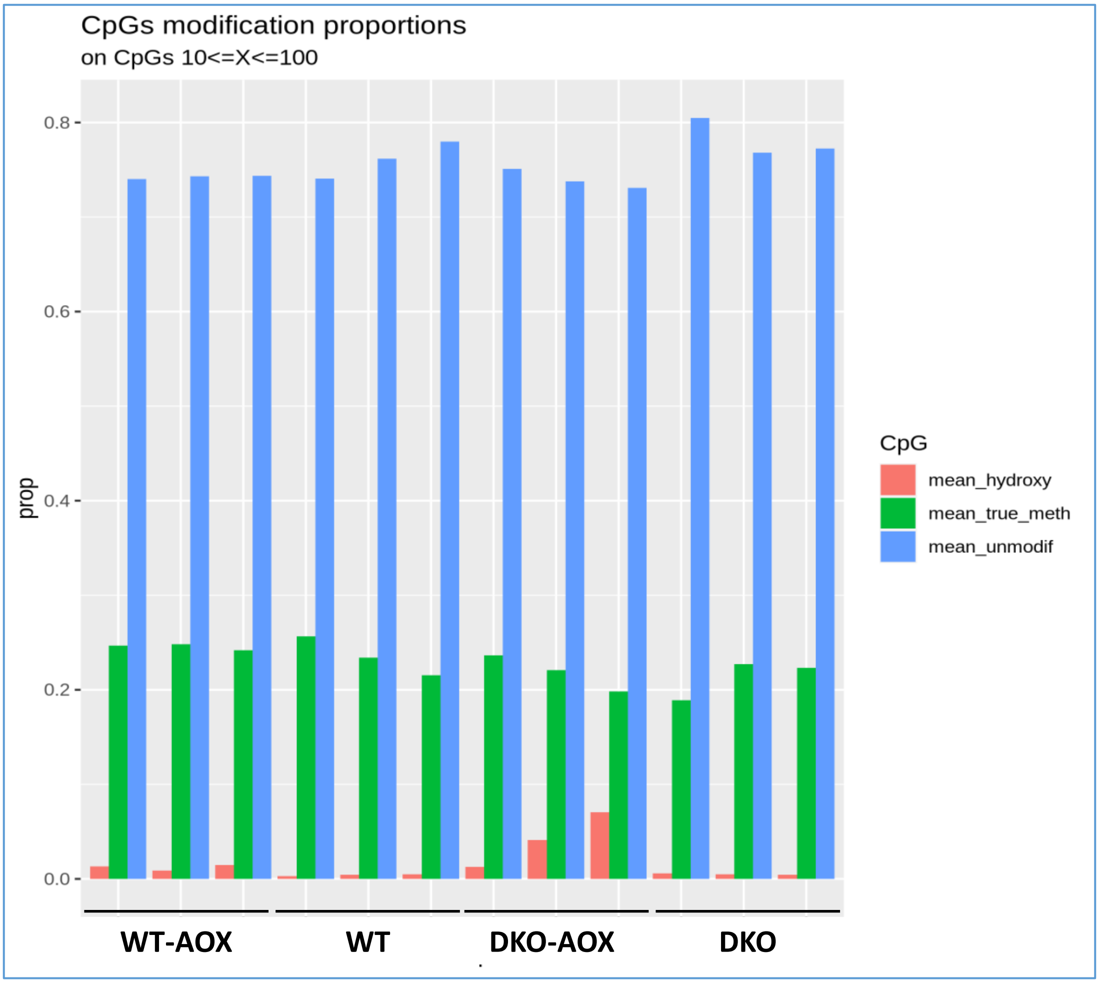

Figure S2: Analysis of individual sequencing data.

(A) Total number of CpG sites within the depth range of 10X to 100X, obtained by RREM-Seq for 5mC study or by RREhM-Seq for 5hmC study. This number exceeds 1.5 million for all samples in each category and is relatively uniform. **(B)** Global proportions of unmethylated, methylated and hydroxymethylated cytosines, in the depth range of 10X to 100X, for each individual. Data show no differences except for the DKO-AOX group, which exhibits hyperhydroxymethylation.

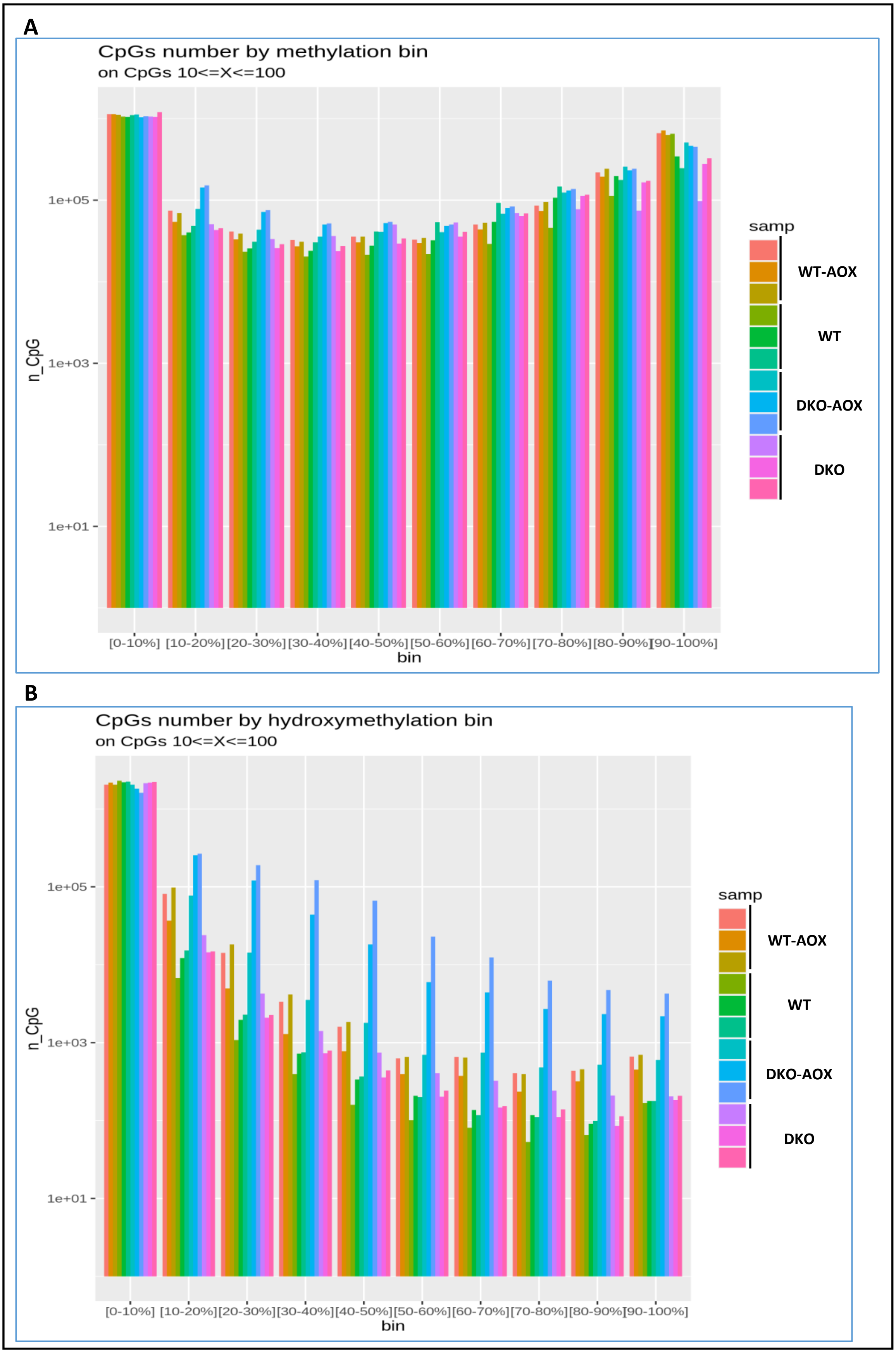

Figure S3. Methylation and hydroxymethylation status of cytosines.

For each sequenced 5mC **(A)** and 5hmC (**B**), and for each individual, the frequency of the (hydroxy)methylation, expressed in %, has been represented respecting ten % intervals. The number of CpG sites per 10% methylation bin (A) shows relative uniformity among the different groups. The majority of CpG sites fall within the low methylation 0%-10% bin. The majority of 5hmC are also found in the low bin of 0% to 10%. AOX treated individuals show an enrichment for hydroxymethylated CpG sites compared to other groups, with a higher increase observed for DKO individuals

A

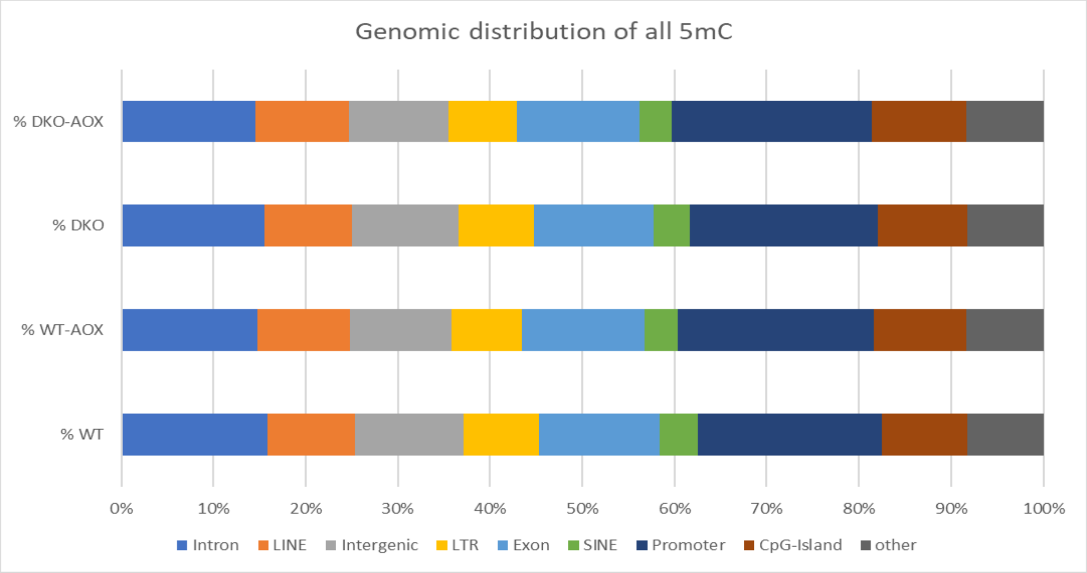

B

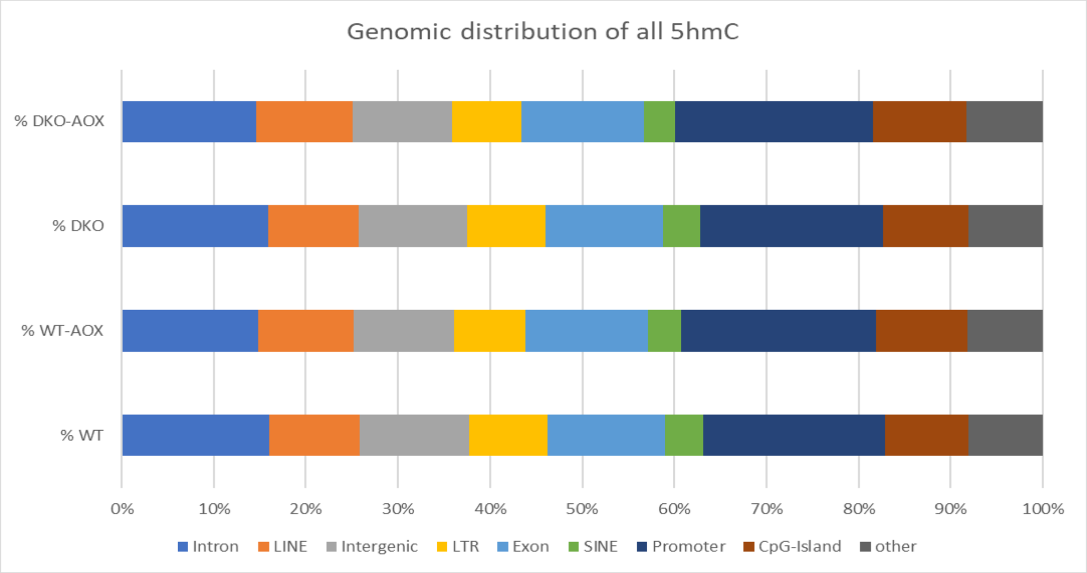

Figure S4. Genomic repartition of the 5mC (A) and 5hmC (B) detected by the RREM-seq. The genomic repartition of the detected 5mC loci **(A)** and 5 hmC loci **(B)** are represented on merge replicates for each condition (in % of the total 5mC detected) and computed with Homer suite.

	Fertilix® Formulation		
**No #**	**Ingredient**	**Dose**	**Unit**
1	Vitamin C (as Calcium Ascorbate)	90,00	mg
2	Folate (as Calcium L-5-methyltetrahydrofolate)	0,60	mg
3	Zinc (from Zinc gluconate)	11,00	mg
4	Selenium (from L-Selenomethionine)	0,06	mg
5	Proprietary Carnitine Blend (Acetyl-L-Carnitine + Propionyl-L-Carnitine)	800,00	mg
6	Proprietary Natural Vitamin E Blend (Tocopherols + Tocotrienols)	150,00	mg
7	Vitamin D3 (as Cholecalciferol)	0,04	mg
8	Lycopene	10,00	mg
9	CoQ10 (as Ubidecarenone)	20,00	mg

Figure S5: Composition of the Fertilix® .

DML					DMR			
Methylation	TOTAL	Hyper	Hypo		Methylation	TOTAL	Hyper	Hypo
DKO vs WT	5995	1194	4801		DKO vs WT	1406	202	1204
DKO-AOX vs DKO	18693	14041	4652		DKO-AOX vs DKO	1643	1323	320
WT-AOX vs WT	10567	7033	3534		WT-AOX vs WT	937	695	242
DKO-AOX vs WT-AOX	7305	2160	5145		DKO-AOX vs WT-AOX	801	171	630
DKO-AOX vs WT	22278	12777	9501		DKO-AOX vs WT	1213	606	607
HydroxyMethylation	TOTAL	Hyper	Hypo		HydroxyMethylation	TOTAL	Hyper	Hypo
DKO vs WT	650	508	142		DKO vs WT	0	0	0
DKO-AOX vs DKO	10775	10642	133		DKO-AOX vs DKO	296	295	1
WT-AOX vs WT	2224	2067	157		WT-AOX vs WT	15	15	0
DKO-AOX vs WT-AOX	4900	4777	123		DKO-AOX vs WT-AOX	103	101	2
DKO-AOX vs WT	12749	12663	86		DKO-AOX vs WT	417	416	1

Table S1. Comparisons of the DML and DMR for 5mC and 5hmC between different groups. DML and MDR count were established with DSS R package using callDML function with parameters delta=0.1, p.threshold=0.001 and callDMR function with parameters delta=0.1, p.threshold=0.05.
